# Successful Aging Thru Financial Empowerment (SAFE)

**DOI:** 10.1093/geroni/igaf122.657

**Published:** 2025-12-31

**Authors:** LaToya Hall, Peter Lichtenberg

**Affiliations:** Wayne State University, Detroit, Michigan, United States; Wayne State University, Detroit, Michigan, United States

## Abstract

Current estimates of financial exploitation (FE) show between 5% and 11% of older adults are victimized yearly. The rapid growth of FE and defrauding of older people impels the creation and evaluation of intervention and prevention strategies. The Successful Aging thru Financial Empowerment (SAFE) program was created in 2017 to provide older adults education, support and assistance to address FE. The SAFE program currently focuses on providing no cost financial coaching/economic advocacy services to older adult victims of FE and the administration of FE prevention intervention. This presentation will cover two recent studies on SAFE program services and outcomes: (1) One study examined the financial coaching/economic advocacy intervention. Program records and baseline and follow-up assessments of 115 SAFE clients were used to detail program dynamics and investigate participant outcomes. Participants reported high levels of program satisfaction and significantly less stress at the six-month follow-up. These findings demonstrate empowerment-centered financial coaching interventions can successfully address FE in older adult populations. (2) The second is a pilot study examining the effect and acceptability of the newly developed FE prevention program. Intake and follow up assessment data of the first 45 participants enrolled was analyzed. Findings support the prediction that financial literacy and financial vulnerability scores after the intervention would show significant improvement from baseline scores. Participants’ ratings after each session, with respect to usefulness and trustworthiness, were extremely positive. The financial exploitation prevention intervention program demonstrated acceptability and a positive effect on reducing vulnerability to financial exploitation.

